# Inflammatory Myofibroblastic Tumor Mimicking a Cavitary Lesion in the Lung: A Case Report and a Comprehensive Literature Review

**DOI:** 10.7759/cureus.29193

**Published:** 2022-09-15

**Authors:** Janani Arunachalam, Haripriya Radhakrishnan, Harsh Patel, Gurleen K Johal, Gnana Deepthi Medarametla, Aaiyat Sheikh, Syed Nazeer Mahmood, Viray Shah, Digantkumar Patel, Nisarg Changawala

**Affiliations:** 1 Internal Medicine, KAP Viswanatham Government Medical College, Tiruchirapalli, IND; 2 Internal Medicine, Shimoga Institute of Medical Sciences, Shivamogga, IND; 3 Family Medicine, Central Jersey Urgent Care, Green Brook, USA; 4 Internal Medicine, Hackensack Meridian Palisades Medical Center, North Bergen, USA; 5 Internal Medicine, NRI Medical College and General Hospital, Guntur, IND; 6 Internal Medicine, Era's Lucknow Medical College and Hospital, Lucknow, IND; 7 Pulmonary and Critical Care Medicine, MedStar Washington Hospital Center, Washington DC, USA; 8 Hospital Medicine, MedStar Good Samaritan Hospital, Baltimore, USA; 9 Medicine, Springfield Memorial Hospital, Springfield, USA; 10 Pulmonology, Lung Center of Nevada, Las Vegas, USA

**Keywords:** long term follow up, alk-1 positive, rare lung disease, malignancy, chest imaging, hemoptysis, spindle-shaped cells, inflammatory myofibroblastic tumor

## Abstract

Inflammatory myofibroblastic tumors (IMTs) are a group of soft tissue neoplasms with a predilection for the lungs and abdominopelvic cavity, characterized by a mixture of fasciitis-like, compact spindle cells, hypocellular fibrous histologic patterns, and distinctive molecular features. Due to their unspecified symptoms and non-specific radiologic presentation, the histopathologic and immunohistochemical analysis of a biopsy specimen is crucial for the diagnosis. We present a case of a 30-year-old man with intermittent hemoptysis diagnosed with a pulmonary IMT. We aim to review the literature regarding its definition, clinical findings, diagnosis, treatment, and prognosis. The treatment for an IMT is based on its location and extent, including complete surgical resection, which has a good prognosis compared to corticosteroids, chemotherapy, radiotherapy, and non-steroidal immunomodulation in patients who are not good surgical candidates. Further investigative studies with larger sample sizes and longer meticulous follow-ups are needed to demonstrate this neoplastic disease's natural history and find appropriate management for it.

## Introduction

Inflammatory myofibroblastic tumors (IMTs) were first described by Brunn in 1937. They represent an extremely rare type of inflammatory pseudotumor that appears most commonly in children and young individuals, with a global prevalence ranging from 0.04% to 0.7%, irrespective of gender or race [1]. IMTs are a group of soft tissue neoplasms formed by spindle-shaped myofibroblasts and inflammatory cells (eosinophils, plasma cells, and lymphocytes) with a predilection for the lungs and abdominopelvic region [2]. Although they account for about 50% of benign pulmonary masses in children, they represent less than 1% of adult lung tumors [2,3]. Unspecified symptoms, such as cough, chest pain, dyspnea, hemoptysis, and non-specific inflammatory symptoms like fever, malaise, and weight loss, are the presenting features most commonly seen in IMTs of the lungs [2]. The radiographic presentation of pulmonary IMTs is also non-specific. They can be solitary or multiple well-circumscribed peripheral lung masses. Because of their similar radiological appearance to other malignant lung masses and aggressive behavior with the invasion of adjacent structures, histopathologic and immunohistochemical analysis of a biopsy specimen is the mainstay for diagnosis [2,4].

Here, we discuss the case of a 30-year-old male who reported hemoptysis and was diagnosed with a pulmonary IMT. The aim of this report is to present a case of an inflammatory myofibroblastic tumor diagnosed by immunochemistry and review the literature on its definition, clinical findings, diagnosis, treatment, and prognosis.

## Case presentation

A 30-year-old man with a history of nephrolithiasis presented to the emergency room with complaints of flank pain and an episode of small-volume hemoptysis (two to three teaspoonfuls of blood a day) before presentation. He denied fever, chills, chest pain, night sweats, or weight loss. The patient reported intermittently experiencing episodes of non-massive hemoptysis for the last six years. There was no known history of tuberculosis or valley fever exposure, and he did not have a history of recent travel.

**Figure 1:**
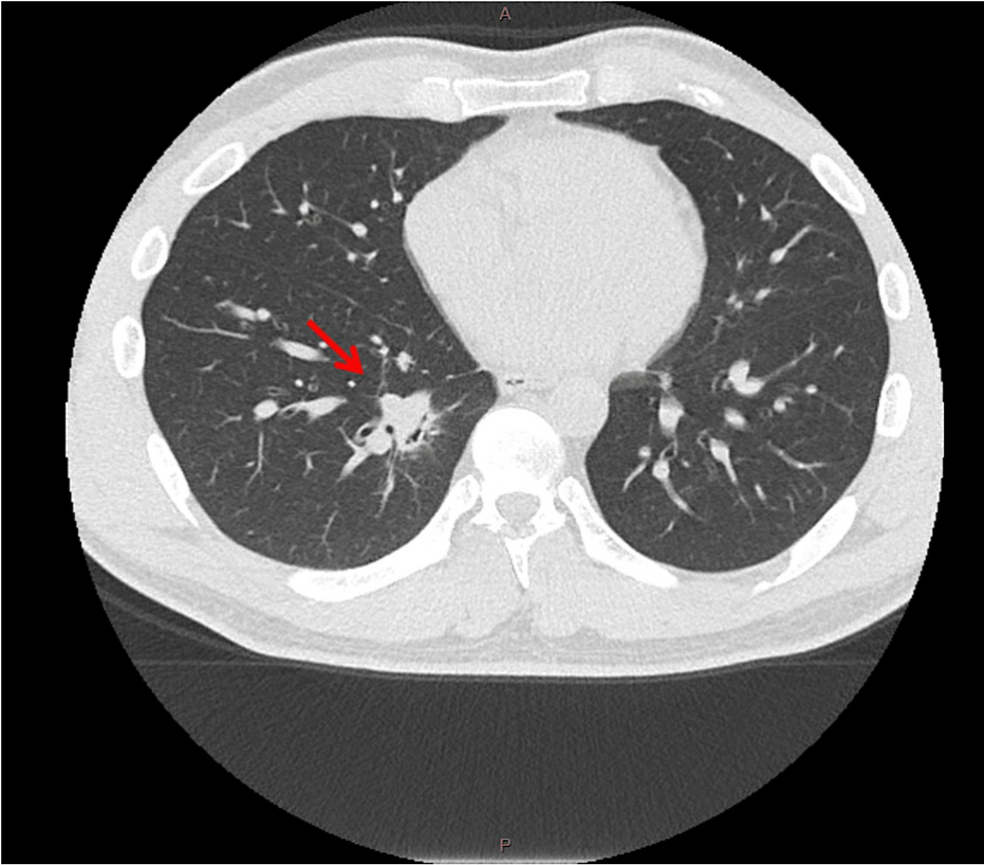
Chest CT image showing a right lower lobe nodule - arising adjacent to the right posterior basal segmental bronchus along with adjacent ground-glass airspace opacities likely reflecting pulmonary hemorrhage

On admission, computed tomography angiography (CTA) of the chest revealed a lesion initially interpreted by radiologists as a cavity. However, it was concluded that the lesion was a solid nodule containing rounded soft tissue mass, with a dilated airway mimicking a cavitary change. It was seen to be arising adjacent to the right posterior basal segmental bronchus along with adjacent ground-glass airspace opacities, likely reflecting pulmonary hemorrhage (Figure 1).

This lesion appeared to have progressed since a prior CT scan was done five months earlier. Differential diagnoses of the lesions included fungal infection, bacterial superinfection of a preexisting right lung cystic lesion, or a neoplasm such as bronchial carcinoid. Bronchoscopy and bronchoalveolar lavage (BAL) of the posterior basal segment of the right lower lobe (RLL) were notable for some fresh blood emanating from the posterior basal segment of the RLL but no endobronchial lesion. A biopsy was not done. BAL cultures were negative for bacteria, fungus, or acid-fast bacilli (AFB).

**Figure 2:**
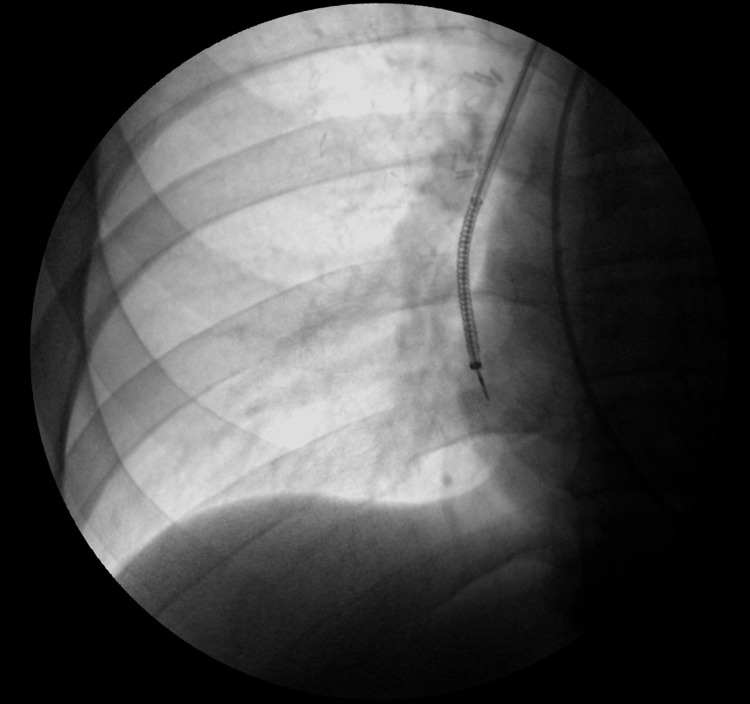
Robot-assisted bronchoscopy with right lower lobe lung nodule transbronchial needle aspiration biopsy using radial endobronchial ultrasound and fluoroscopy

The patient was subsequently discharged home with plans to follow up on an outpatient basis. An Ion protocol CT chest scan was done three weeks later, which showed a right infrahilar mass measuring 2.4 x 1.7 x 2.4 cm in size, showing an increase in size. Three weeks later, a robot-assisted navigational bronchoscopy with radial endobronchial ultrasound (r-EBUS) and fluoroscopy-assisted transbronchial needle aspiration and brushing of the RLL lung nodule were performed (Figure 2).

**Figure 3:**
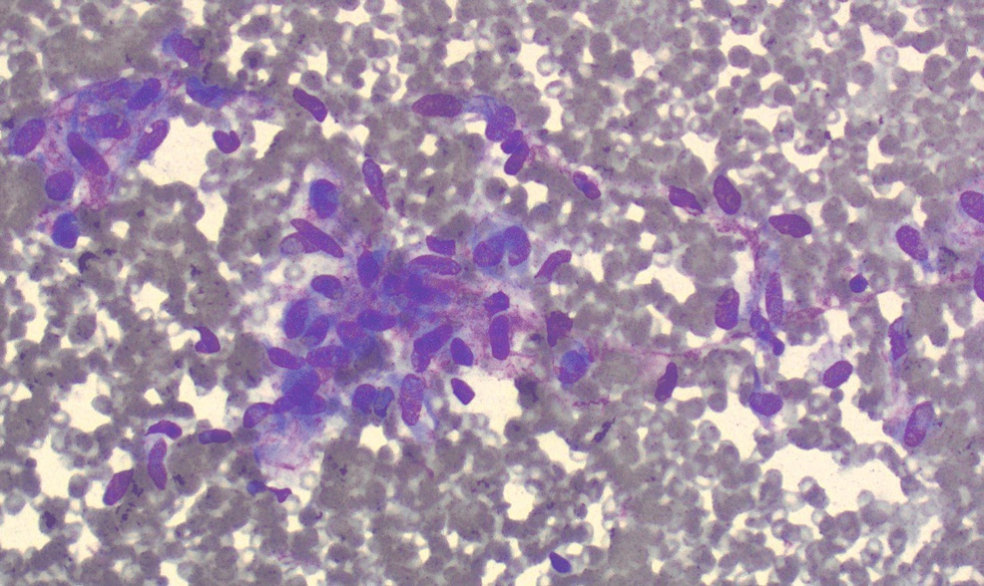
Fine needle aspiration showed clusters of spindle cells with oval and elongated nuclei and fragile cytoplasm

Culture results were negative for bacteria, fungus, or AFB. The fine needle aspiration showed clusters of spindle cells with oval and elongated nuclei and fragile cytoplasm (Figure 3).

**Figure 4:**
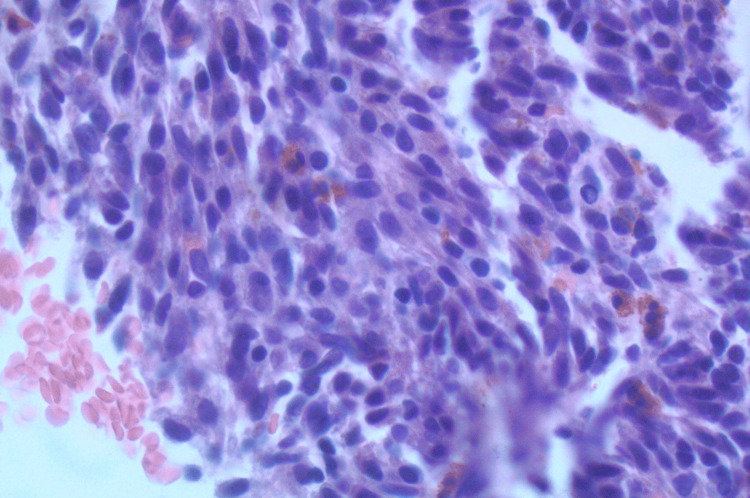
On the cell block, the lesion was composed of spindle cells with inflammatory cells in the background including eosinophils and lymphocytes

On the cell block, the lesion was composed of spindle cells with inflammatory cells in the background including eosinophils and lymphocytes (Figure 4).

**Figure 5:**
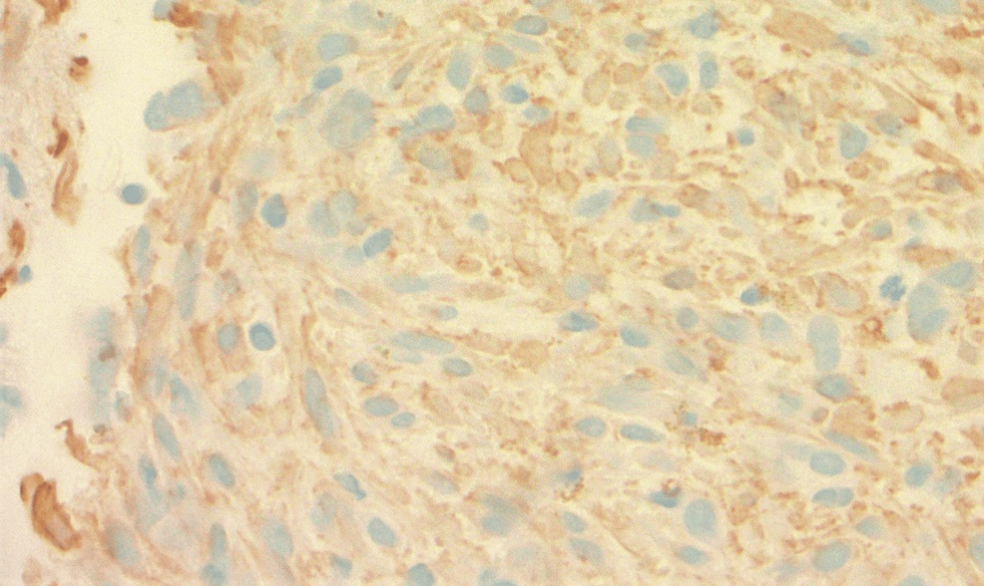
Spindle cells showing cytoplasmic staining with smooth muscle actin

**Figure 6:**
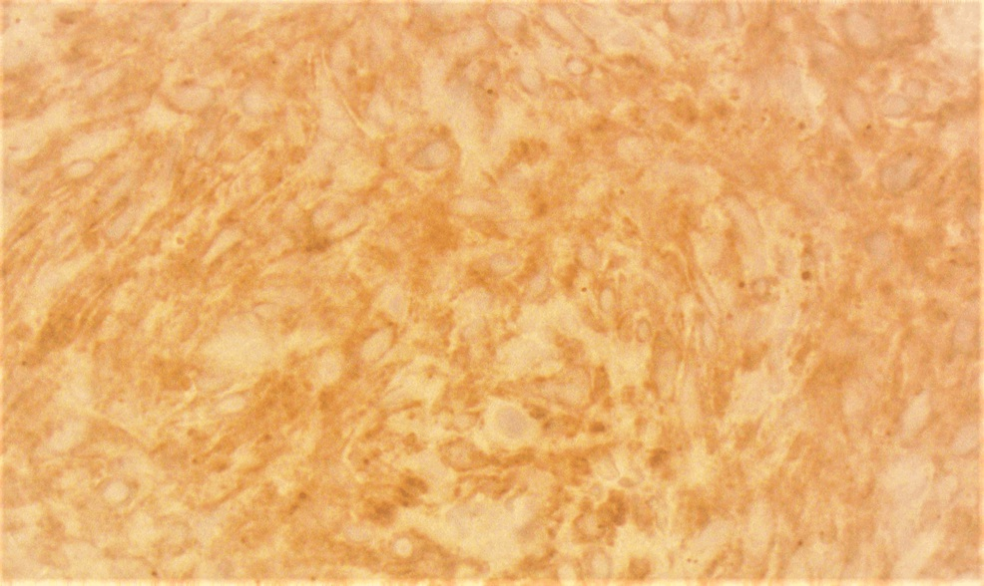
Spindle cells showing cytoplasmic staining with anaplastic lymphoma kinase

On immunohistochemical staining, the spindle cells showed cytoplasmic staining with smooth muscle actin (SMA) and anaplastic lymphoma kinase (ALK) (Figures 5, 6).

The spindle cells did not express S-100, SOX-10, cytokeratin AE1/3, CAM 5.2, CD56, synaptophysin, chromogranin, TTF-1, desmin, and HHV-8. The morphology and immunoprofile together support the diagnosis of an inflammatory myofibroblastic tumor. The patient was referred to an oncologist as well as a thoracic surgeon for further management, and was lost to follow-up.

## Discussion

An inflammatory myofibroblastic tumor is a unique tumor with predominantly benign potential, with a few reports of distant metastasis [5]. It varies significantly in clinical and histological findings, progression, and prognosis. The diagnosis is primarily histological. However, it is challenging to diagnose the disease in such a manner because the histological characteristics of the tumor are similar to other neoplasms, such as low-grade sarcoma and lymphoma, all of which are characterized by infiltration of varying polymorphic cells [6].

IMTs are also difficult to diagnose by imaging, as was demonstrated in this case. The rarity of this tumor, along with its non-preference for a specific bodily location, makes the process of diagnosis arduous. However, IMTs grow and can start invading adjacent organs. Hence, the need for an accurate and speedy diagnosis is of the greatest importance. The rate of growth, as well as the nature of the tumor, can be determined by its associated biological markers [7]. It was found that tumors with absent ALK expression are of a more aggressive nature, with higher mortality rates due to distant metastases. The absence of p53 nuclear expression was also noted in 75% of the IMTs of metastatic nature in the same study [8].

When involving the lungs, the tumor presents with non-specific respiratory and systemic symptoms, and therefore chest imaging is crucial in identifying lung lesions early. Patients with a similar presentation require a biopsy of the lung lesions either via CT-guided biopsy or bronchoscopy with endobronchial ultrasound and fine needle aspiration for a histological diagnosis. As discussed above, histological examination alone is inconclusive. To differentiate it from various benign and malignant tumors conclusively, immunohistochemistry for ALK expression, fluorescence in situ hybridization (FISH), and next-generation sequencing (NGS) are assistive [8].

The treatment for IMT depends mainly on the site and extent of the tumor. Complete surgical resection is associated with a better long-term prognosis. However, lobectomy and pneumonectomy are preferred over wedge resection or endobronchial resection, as these decrease the risk of local recurrence and metastasis more effectively [8]. A good prognosis was seen in patients undergoing complete resection of the pulmonary inflammatory myofibroblastic tumor (PIMT), with 5- and 10-year survival rates of 91% and 77%, respectively [9]. The recurrence rate of PIMTs can be up to 60%, but complete resection reduces it to less than 2% [9,10]. Although the recurrence rate after surgical management remains low, a long-term follow-up is advised as cases of recurrence have been reported even after 11 years [1,9,11]. Corticosteroids can be used when the mass has invaded adjacent structures or has multifocal involvement, making it unresectable. If ALK-1 is positive, like in our patient, crizotinib, a competitive inhibitor of ALK tyrosine kinase, has been found to be effective in some cases [12].

Chemotherapy, radiotherapy, and non-steroidal immunomodulation have been attempted in patients who were not good candidates for surgery. However, these treatment methods were found to be ineffective against this aggressive tumor [13]. Table 1 shows a comprehensive list of IMT case reports by other authors.

**Table 1:** A comprehensive list of case reports published by other authors PET, positron emission tomography; SMA, smooth muscle actin; APA, argon plasma coagulation; ALK, anaplastic lymphoma kinase; CXR, chest X-ray; EIMS, epithelioid inflammatory myofibroblastic sarcoma; RUL, right upper lobe; RLL, right lower lobe; EMA, epithelial membrane antigen; CK-LMW, low molecular weight cytokeratin; CK-HMW, high molecular weight cytokeratin

Author, year	Age (years)/sex	Signs and symptoms	Location	Immunostain profile	Treatment	Outcome
Sagar et al., 2018 [3]	51/female	Recurrent pneumonia over 6 months	Left lower lobe mass with endobronchial extension	Positive - caldesmon, desmin, SMA ALK-1; negative - keratin S-100	Sleeve resection	Stable disease at 2.5 months from diagnosis, lost to follow-up afterwards
Sagar et al., 2018 [3]	45/female	Shortness of breath, dysphagia for 6 months	Tumor extending through the length of trachea	Positive- desmin SMA; negative - cytokeratin, S-100, CD117, HMB-45, CD35, CD23 ALK-1	Celecoxib, steroids and methotrexate	Stable disease at 8 months from diagnosis, lost to follow-up afterwards
Sagar et al., 2018 [3]	70/male	Cough, wheeze, hemoptysis for 2 years	Mediastinal mass causing a collapse of the left mainstem due to mass effect	Positive - P-catenin, SMA; negative - C-kit, cytokeratin, CD34, S-100	Surveillance done for 2 years followed by debulking of the tumor, with radiotherapy	Stable disease at 2 years post-treatment, lost to follow-up afterwards
Sagar et al., 2018 [3]	43/female	Recurrent, intermittent hemoptysis for 2 years	Left hilar mass		Pneumonectomy	Alive, stable disease at 4 years post-diagnosis
Sagar et al., 2018 [3]	48/female	Dry cough for 2 weeks	Right infrahilar mass	Positive - desmin, FXIIIa, SMA, CD68; negative - S-100 keratin	Right lower lobectomy	Alive, stable disease at 3 months from diagnosis, lost to follow-up afterwards
Sagar et al., 2018 [3]	43/female	Recurrent respiratory infection for 2 years. Rib and arm pain, dry cough, dyspnea	Right upper lobe mass with endobronchial extension	Positive - SMA, vimentin; negative - S-100, CD34, keratin	Right upper lobectomy and APC with electrocautery. Snare for recurrent endobronchial disease performed 9 months after initial treatment	Recurrent disease 9 months after surgery; lost to follow-up afterwards
Sagar et al., 2018 [3]	18/female	None	Left-sided para-aortic mass	Positive - vimentin, SMA; negative - ALK-1, S-100, CD35, CD21, CD23	Neoadjuvant chemotherapy (MTX and vinblastine), pneumonectomy XRT	No evidence of recurrence of disease 10 years post-treatment
Yoon et al., 2013 [14]	25/male	None, routine chest X-ray	Right paratracheal space (8 x 6 x 5 cm)		Resection by thoracotomy	
Ohba et al., 2021 [15]	22/female	Bloody sputum and stridor	Posterior wall of trachea	Positive - ALK, S-100, CD1a, CD68, CD31, β-catenin (cytoplasmic), MIC2/CD99 (cytoplasmic); negative - SMA	Transtracheal procedure for the endotracheal tumor	
Ponzoni et al., 2020 [16]	15/male	Persistent fever	Mass in the main pulmonary artery (52 x 25 mm)	Positive - vimentin, alpha-SMA, ALK mutation; negative - calponin	Surgical resection initially. After 5 months, presented with hemoptysis. Angio-CT scan revealed mass in the right pulmonary artery with multiple distal perfusion defects. Suspected thrombotic and secondary lesions; tumor completely resected from the branches of the arteries	Two months after surgery, control CT and PET scan done; a clear main pulmonary artery, stable parenchymal pulmonary lesions, and distal defects of perfusion in the lobar pulmonary arteries, with a possible residual mass in the right pulmonary artery
Tsuchiya et al., 2018 [17]	54/female	Routine CXR	Hilar mass compressing the anterior segmental bronchus of left lung (18 x 9 mm)	Positive - ALK gene; partially positive - desmin, alpha-SMA	Left upper division segmentectomy through an anterolateral thoracotomy	Stable disease 2 years post-surgery
Fu et al., 2015 [18]	21/male	General fatigue, and rapid weight loss, in the setting of a past medical history of EIMS with multiple bone metastases	Left lower lobe of lung without signs of neighboring pleura invasion (10 x 8 cm)	Positive - ALK; negative - pan-CK (AE1/AE3), SMA, HHF35, myogenin, Myo D1, S-100, HMB45, Melan A, synaptophysin, CD34, CD68 and CD30	Total resection of the lung mass	3 months after lung mass resection, multiple bone metastases and intraspinal mass were found by PET. A second surgical treatment was performed to remove the intraspinal lesion.
Oztuna et al., 2013 [19]	20/female	Hoarseness, cough (without sputum), exertional dyspnea for 1 year, and a recent wheezing attack	Diagnostic flexible bronchoscopy revealed tracheal stenosis approximately 2 cm below the vocal cords, with an irregular mucosal appearance	Positive - desmin in smooth muscle cells, and trichrome in collagen tissue; negative - S-100	Rigid bronchoscopy for mechanical dilation and resection was performed in both the trachea and bronchial system	Asymptomatic at 6-month follow-up, with normal thorax CT and pulmonary function test
Morar et al., 2012 [20]	28/male	Recurrent mild hemoptysis for 8 weeks	Coin lesion in the posterior segment of the RUL of the lung (1.5 x 2 cm)		Exploratory thoracotomy and wedge resection of the RUL mass	Remained in remission 3 years post-surgery
Zhang et al., 2021 [21]	80/male	Fatigue for 2 months	Pulmonary mass in the RLL and pleural effusion within the right thoracic cavity (53 x 76 mm)	Positive - EMA, vimentin, actin and Ki-67; negative - ALK protein, desmin, SMA, CD34, CD117, DOG-1, P53, pan-cytokeratin, CK-LMW, CK-HMW and S-100 protein	Could not undergo surgery due to his advanced age, anemia, and hypoproteinemia; three rounds of percutaneous microwave ablation	No evidence of recurrence nearly 3 years later

## Conclusions

Inflammatory myofibroblastic tumors are an uncommon subgroup of benign soft tissue neoplasms for which an exhaustive clinical exam, as well as investigative procedures, must be conducted first. In short, inflammatory myofibroblastic tumors may be considered per exclusion. Competitive inhibitors of ALK tyrosine kinase, such as crizotinib, may be used with a variable response if ALK-1 markers are positive. Because of the rarity and non-specific presentation of the pulmonary inflammatory myofibroblastic tumor, as seen in our case, we strongly suggest including the disease as a differential diagnosis when patients present with unclassifiable respiratory signs and symptoms. Histopathological evaluation is necessary for diagnosis and should be done early, given the aggressive nature of the tumor. For higher diagnostic rates and favorable outcomes, further investigative studies with larger sample sizes and longer follow-ups are needed to establish the natural history of this neoplastic disease and discover appropriate medical management.
